# Performance Analysis and Optimization of a PBDB-T:ITIC Based Organic Solar Cell Using Graphene Oxide as the Hole Transport Layer

**DOI:** 10.3390/nano12101767

**Published:** 2022-05-22

**Authors:** Ghazi Aman Nowsherwan, Abdul Samad, Muhammad Aamir Iqbal, Tauqeer Mushtaq, Ameer Hussain, Maria Malik, Sabah Haider, Phuong V. Pham, Jeong Ryeol Choi

**Affiliations:** 1 Centre of Excellence in Solid State Physics, University of the Punjab, Lahore 54590, Pakistan; ghaziaman.pu@gmail.com (G.A.N.); abdulssamad0@gmail.com (A.S.); tauqeer.raja.333@gmail.com (T.M.); ssprameerhussain@gmail.com (A.H.); mariamalikc@gmail.com (M.M.); 2 School of Materials Science and Engineering, Zhejiang University, Hangzhou 310027, China; 3 School of Chemistry, University of the Punjab, Lahore 54590, Pakistan; sabahaider70@gmail.com; 4 Hangzhou Global Scientific and Technological Innovation Center, Zhejiang University, Hangzhou 310027, China; phuongpham@zju.edu.cn; 5 Department of Nanoengineering, Kyonggi University, Suwon 16227, Korea

**Keywords:** graphene oxide, PBDB-T:ITIC, hole transport layer, PEDOT:PSS, SCAPS, solar cell

## Abstract

The hole transport layer (HTL) in organic solar cells (OSCs) plays an imperative role in boosting the cell’s performance. PEDOT:PSS is a conventional HTL used in OSCs owing to its high design cost and instability issues. It can be replaced with graphene oxide to increase the cell performance by overcoming instability issues. Graphene oxide (GO) has gained popularity in recent years for its practical use in solar energy due to its remarkable mechanical, electrical, thermal, and optical properties. This work uses SCAPS-1D to examine the results of graphene oxide (GO)-based organic solar cells by giving a comparison between the performance of absorber layers and a GO-based HTL to see which absorber material interacts more strongly with GO. The absorber layer PBDB-T:ITIC paired with GO as HTL outperforms the other absorber layers due to its better optical and electrical characteristics. Numerical simulations are performed within the SCAPS software at various absorber layer thicknesses, defect densities, and doping values to assess the influence on device performance and efficiency. After cell optimization, the best efficiency of an improved OSC is found to be 17.36%, and the outcomes of the simulated OSC are referenced to the results of the experimentally implemented OSC. These results provide a possible future direction for developing GO-based OSCs with higher efficiency.

## 1. Introduction

Organic solar cells are classified as third-generation solar cells not because of the year they were invented but because of the material arrangement used in their construction. Dye-synthesized solar cells (DSSCs), polymer-based solar cells, and, in some instances, perovskite solar cells are all being investigated in specialized sectors such as solar energy. Organic photovoltaic devices have received increased attention during the last decade because of their potential uses as flexible, renewable, and nonconservative energy sources. The primary advantages of solar cells include their low cost, mechanical flexibility, light weight, high efficiency, and the ability to be manufactured at low temperatures, among other things [1,2,3,4,5,6]. Calvin developed the first organic solar cell based on magnesium phthalocyanine (MgPc) in 1958 and achieved a 200 mV open-circuit voltage [7]. Later, at 690 nm, 0.01% power conversion efficiency (PCE) was reported using an Al/MgPc/Ag cell [8]. The efficiency of a device using polyacetylene as the absorber layer was 0.3 percent, and the open-circuit voltage (Voc) was 0.3 V [9]. Tourillon et al. suggested an Al/poly (3-nethyl-thiophene)/Pt organic solar cell with a Voc and an external quantum efficiency (QE) of 0.4 V and 0.17% [10].

The poor power conversion efficiencies (PCE) and low quantum efficiencies (QE) of single-layer OSC structures prompted scientists to develop two-layer bilayer structures between the electrodes. As per the existing literature [11,12,13,14], the bilayer heterojunction (BHJ) OSC is the most extensively employed because it provides a better path for separation and transportation of charge carrier materials, which assists in achieving high productivity. Sariciftci et al. fabricated a C60/MEH-PPV bilayer OSC that yields an FF of 0.48% and a PCE of 0.04% [15]. Later on, PPV/C60-based organics show a QE of 9%, a PCE of 1%, and a fill factor of 0.48 [16]. Halls et al. created an organic cell with an electron donor layer (EDL) of bis(phenethylamine) perylene, yielding an external QE peak of 6% and a PCE peak of 1% [17]. Based on these core findings, energy conversion efficiency (PCE) increased dramatically after 2000, increasing from 1 to 12% by 2013. According to a recent study, the photocurrent density of organic solar cells (OSCs) generated by solar radiation is nearing 20 mA/cm^2^, equivalent to that of inorganic solar cells [6]. In 2013, the reported efficiency of OSC was 2.5%. The current improvement increased PCE by 18% in 2020 due to recent developments, owing mainly to the introduction of these unique nonfullerene acceptors (NFA) [18,19]. NFA acceptors are not associated with stability issues and play a key role in enhancing the performance of organic solar cells (OSC) [20,21,22,23,24,25,26,27,28]. PEDOT:PSS is widely used as a hole transport material (HTM) in traditional OSC [29,30] because it provides better hole transportation and has high transmission. On the other hand, it has some limitations (acidic and hygroscopic nature), which degrade the device’s performance. For organic photovoltaic (PV) devices, an increase in conductivity and mobility of charge carriers towards the corresponding electrodes enhances device performance by lowering the recombination at interfaces. Recently, thin films of graphene oxide have been employed as a hole transport medium in OSC, and the results are significantly better than devices manufactured with PEDOT:PSS [31]. In a polymer-based solar cell, Liu [32] achieved the maximum PCE with reduced GO as an HTM compared to the other HTMs. In a heterojunction solar cell, Dan et al. utilized a hybrid bilayer of GO and PEDOT:PSS as hole extraction material and significantly improved device performance [33]. Rafique et al. [34] created a solution-based OSC with a GO and PEDOT:PSS composite bilayer for hole extraction and discovered a PCE of 5.24%. Furthermore, Ozcan et al. observed an increase in productivity of devices by more than 2% when employing GO with PEDOT:PSS [35].

For many years, the SCAPS-1D model has been widely used in thin-film solar cell research to examine the impact of material properties and device designs on thin-film solar cell performance. K.S. Nithya created an NFA-OSC using the SCAPS-1D software. They used CuI as a hole transport layer (HTL), claiming it is more efficient than traditional structures. Under optimal conditions, their device achieves an efficiency (PCE) of 15.68% [36]. Aziz and colleagues modelled and discovered that the NFA bulk heterojunction (BHJ) solar cell performed very well under optimization with few parameters. With the assistance of nonfullerene acceptors, they achieved a PCE of 14.25% [37]. Sharma and colleagues used SCAPS-1D to make a nonfullerene OSC, with CuSCN functioning as the hole transport layer (HTL). After tweaking the parameters, they achieved a power conversion efficiency of 20.36% [38]. Farah et al. utilized SCAPS-1D to evaluate the efficiency of DSSC at a variety of high-temperature settings. According to their findings, the CuI as an HTL outperforms the other two HTLs in performance and outcome [39]. Eri et al. used graphene oxide (GO) as the HTM in a perovskite solar cell, and they obtained better power conversion efficiency than Spiro-MeOTAD [40]. Shobih et al. [41] used SCAPS-1D simulation to investigate the effect of parameters to determine which factors should be optimized to improve device performance. They achieved a maximum PCE of 16.51%, with GO as HTL [41]. The SCAPS-1D is an advanced simulating tool for the design and analysis of high-performance PV cells, including CIGS, cadmium telluride (CdTe), perovskite solar cell (PSC), and CZTS [42,43,44,45,46,47].

OSCs are considered to encourage renewable energy sources as potential alternatives to inorganic PV cells. In this research, we have replaced conventional HTL PEDOT:PSS with graphene oxide as PEDOT:PSS to overcome the stability problems of HTL due to its acidic nature. Graphene oxide does not have stability issues and yields better outcomes than PEDOT:PSS. We also explored the performance of different groups of active layers paired with graphene oxide and different OSC parameters that play an essential role in boosting its performance. The results are also compared with the experimental data reported in other literature. This work is mainly aimed at determining the most acceptable parameters for graphene oxide-based organic solar cells with an improved device efficiency.

## 2. Numerical Modeling of Device

### 2.1. Approach and Design

SCAPS (version 3.3.07), which was developed at the Department of Electronics and Information Systems (ELIS) of the University of Gent, Belgium, has been used to model and simulate the devices in the various segments [48]. The application is divided into many panels, allowing the user to adjust settings and make judgments about the output. This software package is based on Poisson and continuity differential equations for holes and electrons. Various iteration techniques are used to resolve continuity differential equations with Poisson differential equations, which are the underlying concept of this application [48,49]. These equations can be mathematically written as:

(1) $$\frac{d}{\mathrm{dx}}\left(\epsilon \left(x\right)\frac{d\varphi }{\mathrm{dx}}\right)=q\;\left[p\;\left(x\right)-n\;\left(x\right)+N_{d^{+}}\left(x\right)-N_{a^{-}}\left(x\right)+p_{t}\left(x\right)-n_{t}\left(x\right)\right]$$

(2) $$\frac{{\mathrm{dp}}_{n}}{\mathrm{dt}}=G_{p}-\frac{p_{n}-p_{n0}}{\tau_{p}}-p_{n}{µ}_{p}\frac{\mathrm{dE}}{\mathrm{dx}}-{µ}_{p}E\frac{{\mathrm{dp}}_{n}}{\mathrm{dx}}+D_{p}\frac{d^{2}p_{n}}{{\mathrm{dx}}^{2}}$$

(3) $$\frac{{\mathrm{dn}}_{p}}{\mathrm{dt}}=G_{n}-\frac{n_{p}-n_{p0}}{\tau_{n}}+n_{p}{µ}_{n}\frac{\mathrm{dE}}{\mathrm{dx}}+{µ}_{n}E\frac{{\mathrm{dn}}_{p}}{\mathrm{dx}}+D_{n}\frac{d^{2}n_{p}}{{\mathrm{dx}}^{2}}$$

where ϵ= dielectric constant, q= electron charge, G= rate of generation, D = coefficient of diffusion, φ = electrostatic potential, E = electric field, ${µ}_{n}$ = electron mobility, ${µ}_{p}$ = hole mobility, $p_{n}$-$p_{n0}$ = difference of hole density in n-type region, p(x) = allowed concentration of holes, n(x) = allowed concentration of electrons, $p_{t}\left(x\right)$ = captured holes, $n_{t}(x$) = captured electrons, $N_{d^{-}}$ = ionized doping concentration of donor, ${\mathrm{Na}}^{+}$= Ionized doping concentration of acceptor, x= thickness, $\tau_{p}$ = life time of hole, $\tau_{n}$ = life time of electron, and $n_{p}$-$n_{p0}$ = Difference of electron density in p-type region.

**Figure 1:**
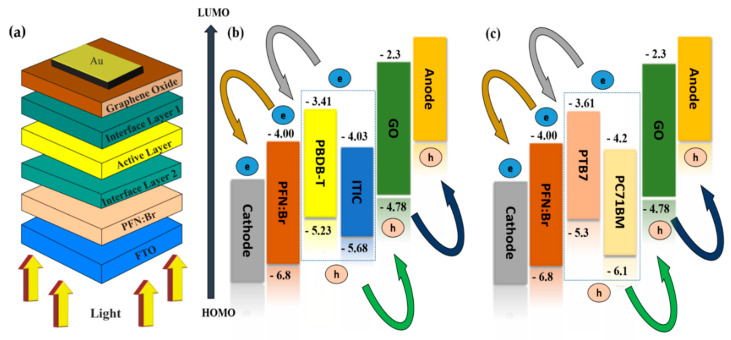
(**a**) Schematic representation of the device architecture; (**b**) HOMO and LUMO band diagram for PBDB−T:ITIC−based OSC; and (**c**) HOMO and LUMO band diagram for PTB7:PC71BM−based OSC.

The adopted bulk heterojunction structure is an organic PV cell structure in which the cell comprises an active layer (PBDB-T:ITIC), hole transport layer (GO), electron transport layer (PFN:Br), transparent conducting oxide (FTO), and back contact (Au), as shown in Figure 1a. Furthermore, the illustrations of the HOMO and LUMO band diagrams of the designed structures of OSC are shown in Figure 1b,c.

### 2.2. Parameters Used in Simulation for Device

The whole set of simulation parameters for the designed layers was selected from the literature published in its entirety in [36,37,40,41,50,51,52,53,54,55]. Numerous material properties must be addressed before simulation, including the donor and acceptor density (N_A_, N_D_), electron and hole mobility (µ_n_, µ_p_), etc. It is critical to consider the individual properties of each material together with the active material, HTL, ETL, and the contact configuration. Each of the critical simulation parameters used in this simulation has been summarized in Table 1 and Table 2.

To facilitate device modeling, absorption coefficient data from several literature works [56,57,58,59,60] has been added to the absorption interpolation model in SCAPS. This device model comprises two interface defect layers, denoted by IL1 (GO/Absorber Layer) and IL2 (Absorber Layer/PFN:Br), to make the device model more realistic. The device modeling technique used the AM1.5G spectrum and operated at a temperature of 300 K. All operational point settings, including parameters, have been reset to their initialization. The voltage range is set from 0 volts to 1.0 volts for scanning. The above parameters have been used to perform all of the simulations throughout this study.

## 3. Results and Discussions

### 3.1. Comparison between Active Layers

This study used two different absorber materials with donor and acceptor components associated with graphene oxide (GO) as hole transport layers (HTL) for organic solar cells (OSC). These materials exhibit more delicate optoelectronic properties due to their high absorption coefficient, charge transfer, and optical conductivity. This research suggests that PBDB-T:ITIC with GO as HTL outperforms other active layers.

**Figure 2:**
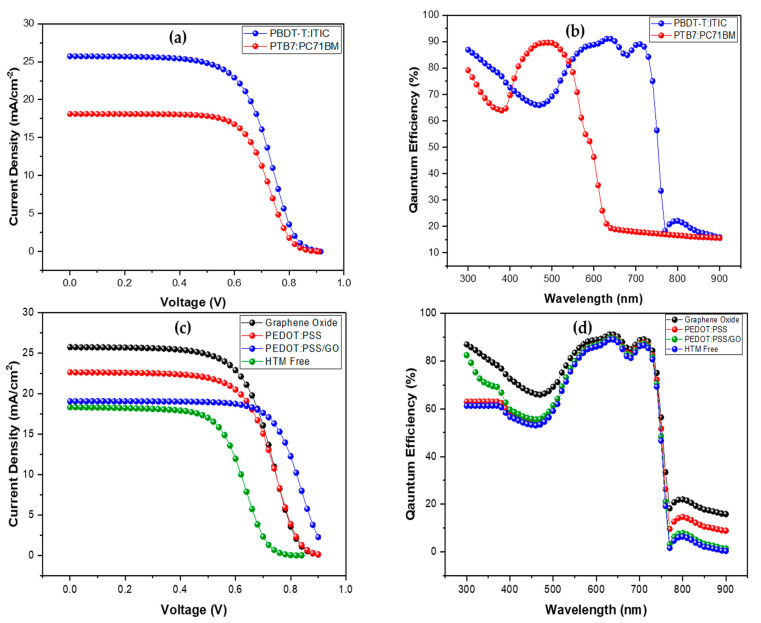
(**a**) Short-circuit current density comparison of different designed absorber layers; (**b**) quantum efficiency comparison of different designed absorber layers; (**c**) short-circuit current density comparison of different designed HTM layers; and (**d**) quantum efficiency comparison of different designed HTM layers.

The numerical analysis and comparison were performed on various absorber layers of OSC, and the results are depicted in Table 3. Figure 2a,b illustrates the current density-voltage (J-V) and quantum efficiency (QE) curves from their differentiation. That visibly demonstrates that the Jsc and Voc values for PBDB-T:ITIC are relatively higher than for other absorber layers. The slight inflection of the J-V curves beyond 0.8 V could be due to the electrical loss in the light-harvesting, hole transport, and electron transport layers, which corresponds to the existence of series resistance (Rs). It is measured as the negative of the inverse slope of the I−V curve near Voc. Rs is primarily caused by contact-related electrical resistance between the transparent conducting oxide and the metal electrode. PBDB-T:ITIC offers better optical and transport properties like a high charge transfer rate, absorption coefficient, and potential dielectric properties [56,61,62]. Therefore, it exhibits high PCE. The PTB7:PC71BM shows an efficiency (PCE) of 10.07% when utilized as an absorber layer with graphene oxide HTL. All the other effects that are demonstrated in this study to see the performance of OSC have been carried out with the PBDB-T:ITIC as an absorber layer.

### 3.2. Comparison between Different Hole Extracting Layers

HTM is essential to attain high efficiency and stability in the device. PEDOT:PSS is a widely used HTM in OSC. However, due to its acidic nature and hygroscopic nature [63,64,65], it is sometimes not favored. There are many alternatives to PEDOT:PSS, but graphene oxide (GO) is utilized as its alternative in this simulation. All simulation parameters for HTM layers in the structure are carefully chosen from the reported experimental data and different works available in the literature [35,36,37,40,41,66,67].

Figure 2c,d represents the J-V and QE curves for OSC with GO, PEDOT:PSS, and PEDOT:PSS/GO as the HTM. The outcomes of these HTM are listed in Table 3. The OSC in conjunction with HTM PEDOT:PSS increases productivity (PCE) by up to 12.33%. Moreover, PEDOT:PSS/GO exhibits a PCE of 12.34%—nearly equal to PEDOT:PSS—but their current density values differ. PEDOT:PSS/GO has a slightly different J-V curve than the other HTMs because it has distinct values for charge carrier mobilities, conductivity, and bandgap [35,67]. Due to this, it may interact differently with the light-harvesting layer. Additionally, without the HTM device, it gives a PCE of 8.6%. However, GO provides better performance and a high efficiency of 13.74% among all HTM. It shows a superior outcome due to its stronger interaction with the absorber layer and better transportation of holes. As GO offers better optical and electrical properties, the probability of recombination losses and diffusion losses is lower at the absorber and HTM interface. The GO can replace PEDOT:PSS due to its high relative stability, wide bandgap, and high p-type conductivity and hole mobility [68,69,70].

Table 4 summarizes a comparison of bulk heterojunction OSC data obtained from simulations and experiments. It has been analyzed that the simulated device outcomes are close to findings reported in simulated and experimental published studies. Therefore, this work also gives theoretical guidelines for the practical application of OSC via optimizing its parameters for the next-generation OSC.

### 3.3. Impact of Layer Thickness on Cell Performance

#### 3.3.1. Impact of Active Layer Thickness

The active layer of any OSC is critical to the device’s functioning and output. In this study, the active layer thickness was manipulated between 100 and 300 nm, and the associated effect on device outcomes was assessed by maintaining all other factors constant throughout the simulations.

**Figure 3:**
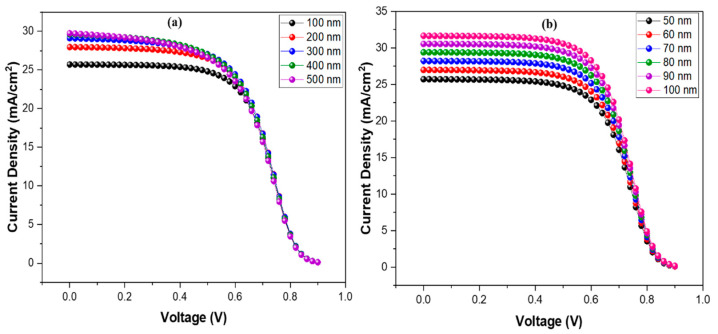
(**a**) Current-density comparison as absorber layer thickness varies; and (**b**) current-density comparison as HTM layer thickness varies.

**Figure 4:**
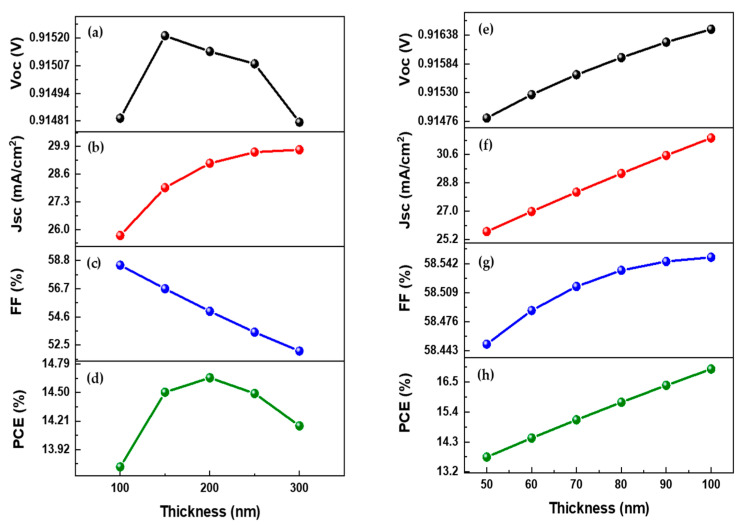
(**a**,**e**) Voc as a function of active layer and HTM layer thickness; (**b**,**f**) Jsc as a function of active layer and HTM layer thickness; (**c**,**g**) FF as a function of active layer and HTM layer thickness; and (**d**,**h**) PCE as a function of the active layer and HTM layer thickness.

The association between the divergence in device results and the active layer’s thickness is seen in Figure 3a and Figure 4a–d. The output parameters Jsc, Voc, and PCE rise dramatically when the absorber layer thickness increases from 100 nm to 200 nm. That is due to a rise in the concentration of electron-hole pairs induced by photon absorption in the absorber layer. Voc and PCE decrease gradually as the device thickness grows from 300 to 500 nm due to increased charge carrier diffusion length and enhanced recombination rate. When the device was 300 nm thick, the maximum Jsc value was 29.75 mA/cm^2^. When layer thickness increases from 100 to 300 nm, the fill factor decreases from 58.45 to 52.06%. The fill factor indicates its capacity to transmit the total available power to the produced electrical load. That might be due to the thick active layer, which raises the cell series resistance.

#### 3.3.2. Impact of HTM Layer Thickness

The hole transport layer heavily influences the output of the OSC. A good selection of HTM allows for improved charge transmission and collection at the electrodes. This study utilizes graphene oxide as an HTL due to its potential optical and electrical properties. Its thickness was changed from 50 nm to 100 nm to observe the effect on output parameters. Jsc and PCE improved greatly when the thickness of the HTM layer was increased, owing to the superior charge transport properties of graphene oxide and better interaction with the absorber layer. The significant effect on output parameters can be visualized in Figure 4e–h, wherein negligible effects can be seen on Voc and FF. The optimized thickness value for HTM can be 100 nm [41], and this value of thickness is very advantageous for making photovoltaic cells more efficient. It also specifies a feasible way toward the efficient application of OSC cells by changing parameters that are highly dependent on the performance and results of OSCs. Figure 3b and Figure 4e–h illustrate the influence on output characteristics.

### 3.4. Impact of Defect Density on Device Performance

#### 3.4.1. Impact of Active Layer Defect Density

The structure and quality of the active layer have a significant impact on the performance and outcome of OSC. The device defect density is crucial in achieving efficient results. If the film quality is poor, the trap density and rate of charge carrier recombination rise, lowering the device performance and outcome.

**Figure 5:**
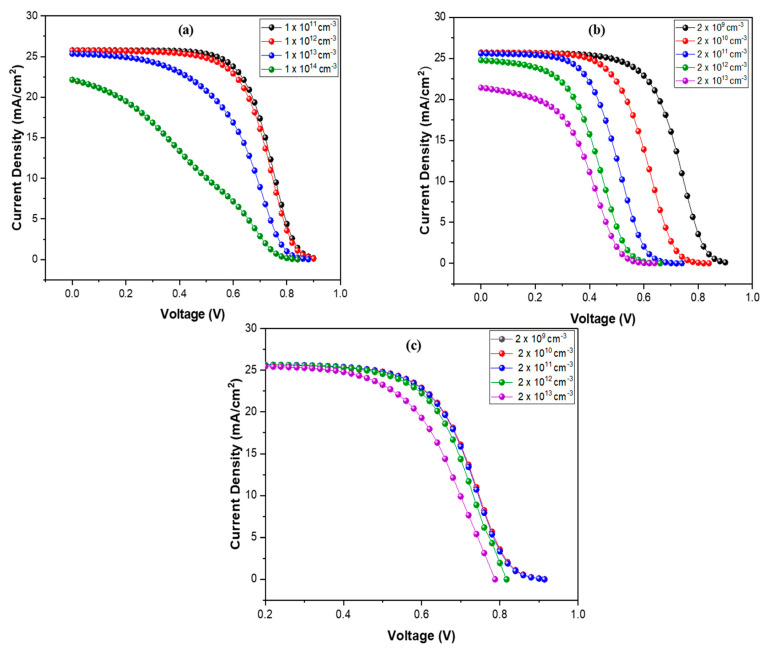
(**a**) Current-density comparison at various defect densities of the absorber layer; (**b**) current-density comparison at various defect densities of IL1; and (**c**) current-density comparison at various defect densities of IL2.

**Figure 6:**
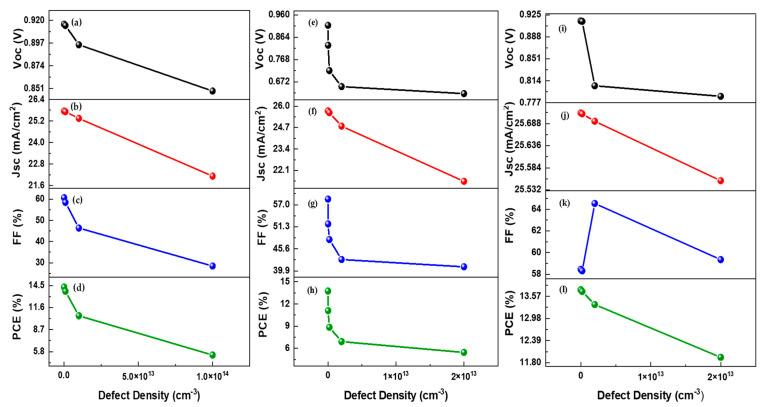
(**a**,**e**,**i**) Voc as a function of active layer, IL1, and IL2 defect density; (**b**,**f**,**j**) Jsc as a function of active layer, IL1, and IL2 defect density; (**c**,**g**,**k**) FF as a function of active layer, IL1, and IL2 defect density; and (**d**,**h**,**l**) PCE versus active layer, IL1, and IL2 defect density.

In this simulation, active layer defects (traps) are varied from 1 × 10^11^ cm^−3^ to 1 × 10^14^ cm^−3^ to analyze their effects on the device performance. The deviation in output parameters with variation in the trap density of the absorber layer is shown in Figure 5a and Figure 6a–d. It is observed that, with the rise in trap density, the output parameters of OSC fall dramatically. The PCE drops significantly from 14.30 to 5.35%, and Jsc drops from 25.75 to 22.13 mA/cm^2^. A surge in defect density leads to a decline in a carrier lifetime, which ultimately reduces the generation rate and promotes recombination.

#### 3.4.2. Impact of Interface Layer Defect Density

Two interface defect layers, IL1 (GO/PBDB-T:ITIC) and IL2 (PBDB-T:ITIC/PFN:Br), are included in this simulation to investigate their impact on the cell’s outcome. The defect densities of the interface layers changed from 2 × 10^9^ cm^−3^ to 2 × 10^13^ cm^−3^, while the rest of the parameters were kept at their default values.

The J-V curves of interfaces for GO/PBDB-T:ITIC0 and PBDB-T:ITIC/PFN: Br at various defect (trap) densities are shown in Figure 5b–c. The deviation in organic solar cell (OSC) result characteristics with varied defect density at interfaces is shown in Figure 6e–l. It can be visualized that low trap density between interfaces is beneficial in enhancing cell outcome because there are few traps and a high growth rate in that case. Voc and Jsc rise when low traps are present, resulting in high PCE and FF. Interfaces with high defect densities generate more capturing states and enhance recombination, reducing device performance. The utmost values of PCE, Voc, and Jsc are 14.74%, 0.91 V, and 25.71 mA/cm^2^, respectively, obtained at the interface defect density value of 2 × 10^9^ cm^−3^ for both interfaces.

### 3.5. Impact of Doping Density on Cell Performance

The doping density of the absorber and HTM layers plays an imperative role in enhancing cell performance. Doping has effects on semiconductor properties as it increases the mobile carrier concentration and decreases mobility because of the motion impedance of the defects produced by the doping atoms [74]. Therefore, the appropriate value for doping density is necessary to yield high outcomes.

#### 3.5.1. Impact of Active Layer Doping Density

**Figure 7:**
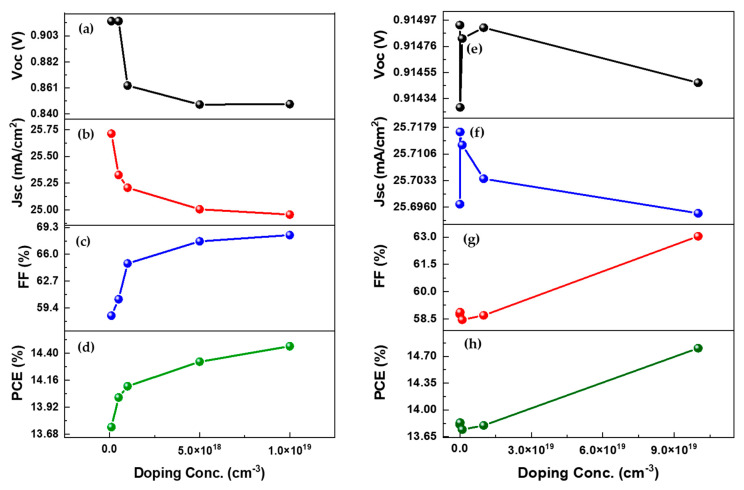
(**a**,**e**) Voc as a function of active layer and HTM layer doping density; (**a**,**e**) Jsc as a function of active layer and HTM layer doping density; (**b**,**f**) FF as a function of active layer and HTM layer doping density; (**c**,**g**) PCE as a function of active layer and HTM layer doping density; and (**d**,**h**) PCE as a function of active layer and HTM layer doping density.

We changed the absorber layer doping from 1 × 10^17^ to 1 × 10^19^ cm^−3^ in this work to see how it affected the device performance, as shown in Figure 7a–d. It is shown that Voc and Jsc decrease because doping weakens the effect of the electric field of the absorber layer. The Jsc value drops from 25.71 mA/cm^2^ to 20.01 mA/cm^2^, and the Voc value drops from 0.91 to 0.85 V. On the other hand, FF and PCE exhibit a continuous increase as doping density improves, which is attributed to uneven charge carrier mobilities. It is determined that the appropriate quantity of doping increases cell performance by lowering free charge carrier recombination.

#### 3.5.2. Impact of HTM Layer Doping Density

This study changed the HTM layer doping density from 1 × 10^16^ to 1 × 10^20^ cm^−3^ to see how it influenced device performance, as shown in Figure 7e–h. It has been analyzed that raising the doping concentration of HTM up to 10^18^ cm^−3^ improves solar cell output performance. This is due to the increased cell conductivity, which causes internal power depletion and series resistance to decrease. The reduction in series resistance increases Jsc, FF, and PCE values [36,37]. FF and PCE improve as doping concentrations rise further, but JSC and Voc drop. It might be due to decreased carrier lifetime and increased recombination rate at the HTM and absorber layer interface. Therefore, the optimized doping density value for the HTM layer could be 1 × 10^17^ cm^−3^.

### 3.6. Optimization of Parameters

The performance of OSC has significantly improved after optimization, and we obtained a promising result with a PCE of 17.38%, which demonstrated that the OSC outcome could be enhanced by appropriately adjusting the parameters. Table 5 summarizes the optimized device parameters along with other performance results.

## 4. Conclusions

In this research, a bulk heterojunction organic solar cell (OSC) device structure has been simulated as FTO/PFN:Br/PBDB-T:ITIC/GO/Au, using SCAPS-1D software, and the performance of two groups of absorber layers has been evaluated along with different HTM layers, in which it is observed that PBDB-T:ITIC performs well because of its superior optoelectronic properties. The device performance at an absorber layer thickness of 100 nm is Voc = 0.9148 V, Jsc = 25.71 mA/cm^2^, FF = 58.45%, and PCE = 13.74%. Furthermore, the effect of the absorber layer thickness, doping density, and defect density on the device performance has been examined, wherein reduced defect density, a medium absorber layer thickness, and an adjusted amount of doping density are depicted as best suited for enhanced photovoltaic properties. The impact of multiple HTMs on OSC performance has also been explored, with GO surpassing the other HTMs. A design of an OSC with a high efficiency of 17.38% is shown along with the simulated results to demonstrate that OSC device performance can be improved by adjusting the device parameters in the near future.

## Figures and Tables

**Table 1:** Material parameters set in simulation.

Parameters	PFN:Br	PBDB−T:ITIC	PTB7:PC71BM	GO
Thickness (nm)	5 [36]	100 [36]	100 [36]	50 [36]
$\mathrm{Acceptor}\;\mathrm{Density}\;\left({\mathrm{cm}}^{-3}\right)$	0	0	0	$10^{18}$ [37]
$\mathrm{Donor}\;\mathrm{Density}\;\left({\mathrm{cm}}^{-3}\right)$	$9\times 10^{18}$ [36]	0	0	0
Effective Density of States for Valence Band (cm^−3^)	10^19^ [36]	10^19^ [36,37]	10^19^ [36,37]	2.2 × 10^18^ [40,41]
Effective Density of States for Conduction Band (cm^−3^)	10^19^ [36]	10^19^ [36,37]	10^19^ [36,37]	1.8 × 10^18^ [40,41]
Bandgap (eV)	2.8 [50]	1.2 [37]	1.1 [51]	2.48 [40,41]
Relative Dielectric Permittivity	5 [36]	3.65 [36]	3.9 [52]	10 [40,41]
Mobility of Electron (cm^2^/V_s_)	2 × 10^−6^ [37]	3.1 × 10^−4^ [36,37]	5 × 10^−4^ [52]	26 [40,41]
Mobility of Hole (cm^2^/Vs)	1 × 10^−4^ [37,50]	3.2 × 10^−4^ [36,37]	5 × 10^−4^ [52]	123 [40,41]
Electron Affinity (eV)	4 [36]	4.03 [36,37]	3.7 [52]	2.3 [40,41]
$\mathrm{Defect}\;\mathrm{Density}\;\left({\mathrm{cm}}^{-3}\right)$	$10^{9}$ [37,50]	$10^{12}$ [36,37]	$10^{12}$ [36,37]	$10^{9}$ [27]

**Table 2:** Device parameters set in the simulation.

Interface Defect Density [36]	
IL1 (ETL/Active Layer) Defect Density IL2 (Active Layer/HTL) Defect Density	$2\times 10^{9}\;{\mathrm{cm}}^{-2}$ $2\times 10^{9}\;{\mathrm{cm}}^{-2}$
**Back Metal Contact Properties** **[54,55]**	
The electron work function of Au Surface recombination velocity of electron Surface recombination velocity of hole	−5.1 eV $10^{5}\;\mathrm{cm}/s$ $10^{7}\;\mathrm{cm}/s$
**Front Metal Contact Properties** **[54,55]**	
The electron work function of TCO Surface recombination velocity of electron Surface recombination velocity of hole	−4.4 eV $10^{7}\;\mathrm{cm}/s$ 10^5^ cm/s

**Table 3:** Device performance with different absorbers and HTM layers.

Absorber Layer	Voc (volt)	$\mathrm{Jsc}\;(\mathrm{mA}/{\mathrm{cm}}^{2})$	FF (%)	PCE (%)
PBDB-T:ITIC	0.9148	25.71	58.45	13.74
PTB7:PC71BM	0.9070	18.12	61.30	10.07
**HTM Layer**				
GO	0.9148	25.71	58.45	13.74
PEDOT:PSS	0.9157	22.63	59.52	12.33
PEDOT:PSS/GO	0.9300	19.07	69.60	12.34
HTM Free	0.8248	18.33	56.90	8.60

**Table 4:** A comparison of bulk heterojunction OSC theoretical and experimental data.

Active Materials	Voc (V)	Jsc (mA/cm^2^)	FF (%)	PCE (%)	Ref.
**Experimental Results**					
PEDOT:PSS/GO/PCDTBT:PC71BM	0.82	10.44	50	4.28	[67]
PEDOT:PSS/GO/PCDTBT:PC71BM	0.85	10.82	57.0	5.24	[34]
PEDOT:PSS/PTB7:PC71BM	0.736	14.89	74.08	5.92	[53]
PTB4/PC71BM	0.70	14.8	64.60	7.1	[71]
PEDOT:PSS/PTB7-Th:PC61BM	0.78	17.66	52.41	7.24	[72]
PEDOT:PSS/PBDB-T:ITIC-OE	0.9562	16.50	69.75	11	[66]
PEDOT:PSS/PBDB-T:ITIC	1.06	16.2	82.95	14.25	[37]
CuI/PBDB-T:ITIC	0.9773	20.15	79.59	15.68	[36]
PBD:PFBSA/PBDB-T:N2200	0.85	24.23	71	16.2	[73]
**Simulation Results**					
GO/PBDB-T:ITIC	0.9148	25.71	58.45	13.74	This study
GO/PTB7:PC71BM	0.9070	18.12	61.30	10.07	This study

**Table 5:** Optimized numerical parameters and performance results.

Parameters	Absorber Layer		HTL	
Thickness (nm)	200		100	
Doping Concentration (cm^−3^)	-		1 × 10^17^	
Defect Density (cm^−3^)	-		1 × 10^10^	
**Device Configuration**	**Voc (V)**	**Jsc (mA/cm^2^)**	**FF (%)**	**PCE (%)**
GO/PBDB-T:ITIC	0.9165	34.19	55.49	17.38
GO/PTB7:PC71BM	0.9017	26.80	57.73	13.95

## Data Availability

Not applicable.
